# Sulforaphane suppresses oral cancer cell migration by regulating cathepsin S expression

**DOI:** 10.18632/oncotarget.24786

**Published:** 2018-04-03

**Authors:** Chang-Tai Chen, Ming-Ju Hsieh, Yi-Hsien Hsieh, Min-Chieh Hsin, Yi-Ting Chuang, Shun-Fa Yang, Jia-Sin Yang, Chiao-Wen Lin

**Affiliations:** ^1^ Institute of Oral Sciences, Chung Shan Medical University, Taichung, Taiwan; ^2^ School of Dentistry, Chung Shan Medical University, Taichung, Taiwan; ^3^ Institute of Medicine, Chung Shan Medical University, Taichung, Taiwan; ^4^ Cancer Research Center, Changhua Christian Hospital, Changhua, Taiwan; ^5^ Graduate Institute of Biomedical Sciences, China Medical University, Taichung, Taiwan; ^6^ Institute of Biochemistry, Microbiology and Immunology, Chung Shan Medical University, Taichung, Taiwan; ^7^ Department of Medical Research, Chung Shan Medical University Hospital, Taichung, Taiwan; ^8^ Department of Dentistry, Chung Shan Medical University Hospital, Taichung, Taiwan

**Keywords:** sulforaphane, cathepsin S, LC3, oral cancer, migration

## Abstract

Sulforaphane has been demonstrated to exert numerous biological effects, such as neuroprotective, anti-inflammatory, and anticancer effects. However, the detailed effects of sulforaphane on human oral cancer cell migration and the underlying mechanisms remain unclear. In this study, we observed that sulforaphane attenuated SCC-9 and SCC-14 cell motility and invasiveness by reducing cathepsin S expression. Moreover, sulforaphane increased microtubule-associated protein 1 light chain 3 (LC3) conversion, and the knockdown of LC3 by siRNA increased cell migration ability. Regarding the mechanism, sulforaphane inhibited the cell motility of oral cancer cells through the extracellular signal-regulated kinase (ERK) pathway, which in turn reversed cell motility. In conclusion, sulforaphane suppress cathepsin S expression by inducing autophage through ERK signaling pathway. Thus, cathepsin S and LC3 may be new targets for oral cancer treatment.

## INTRODUCTION

Oral cancer is a common neoplasm, and its incidence rate ranks fourth among cancer in males in Taiwan. Numerous intensive studies have focused on cancer pathogenesis at the molecular level, providing new therapy options [1]. Multiple pathways are involved in tumor progression, including dysfunction of cell adhesion and degradation of extracellular matrix (ECM) components [2, 3]. It is widely accepted that proteinase degrades the ECM and facilitates malignant progression. Cathepsin S is a member of the lysosomal cysteine family that can degrade ECM elements such as laminin, fibronectin, elastin, and certain collagens [4]. Cathepsin S was reported to play numerous roles in distinct tumorigenic processes, including angiogenesis, metastasis, and apoptosis [5–9]. Moreover, Tsai *et al.* reported that cathepsin S inhibitors could be useful in prevent or delay cancer metastasis [10]. Reports have revealed that inhibition of cathepsin S induces autophagy in various types of human cancer [11, 12]. Autophagy is an evolutionarily conserved catabolic process that maintains cellular and energetic homeostasis by providing nutrients and mitigating cellular damage [13]. Autophagy plays a key role in regulating the balance between cell survival and cell death. Autophagy modulation was suggested to be beneficial for cancer prevention and therapy [14].

Sulforaphane is a natural phytochemical compound derived from cruciferous plants, such as broccoli, cabbage, and cauliflower [15]. Sulforaphane has been demonstrated to exert numerous biological effects, such as neuroprotective, anti-inflammatory, anti-angiogenesis and anticancer effects [16–18]. Studies have reported that sulforaphane eliminates cancer stem cells in various cancers [19, 20]. Several mechanisms have been suggested to clarify these effects, including cell apoptosis [21], angiogenesis [22], and the modulation of cytochrome P-450 enzymes [23]. Myzak *et al.* demonstrated that sulforaphane acts as a histone deacetylase inhibitor in human colon cancer cells by regulating the promoter region of the p21 and BAX genes [24]. In addition, administering sulforaphane to prostate cancer cells induced G2/M cell cycle arrest [25] and reactive oxygen species (ROS) generation [26]. Alongside its chemopreventive properties, sulforaphane was demonstrated to have antimicrobial activity against *Helicobacter pylori*, a strong risk factor for gastric cancer [27]. Moreover, evidence exists regarding the anticancer effect of sulforaphane on other cancer cells, but few studies have examined this effect in oral cancer. Therefore, the mechanism underlying the effects of sulforaphane in oral cancer is worth investigating.

## RESULTS

### Effects of sulforaphane on migration and invasion *in vitro* in SCC-9 and SCC-14 cells

**Figure 1:**
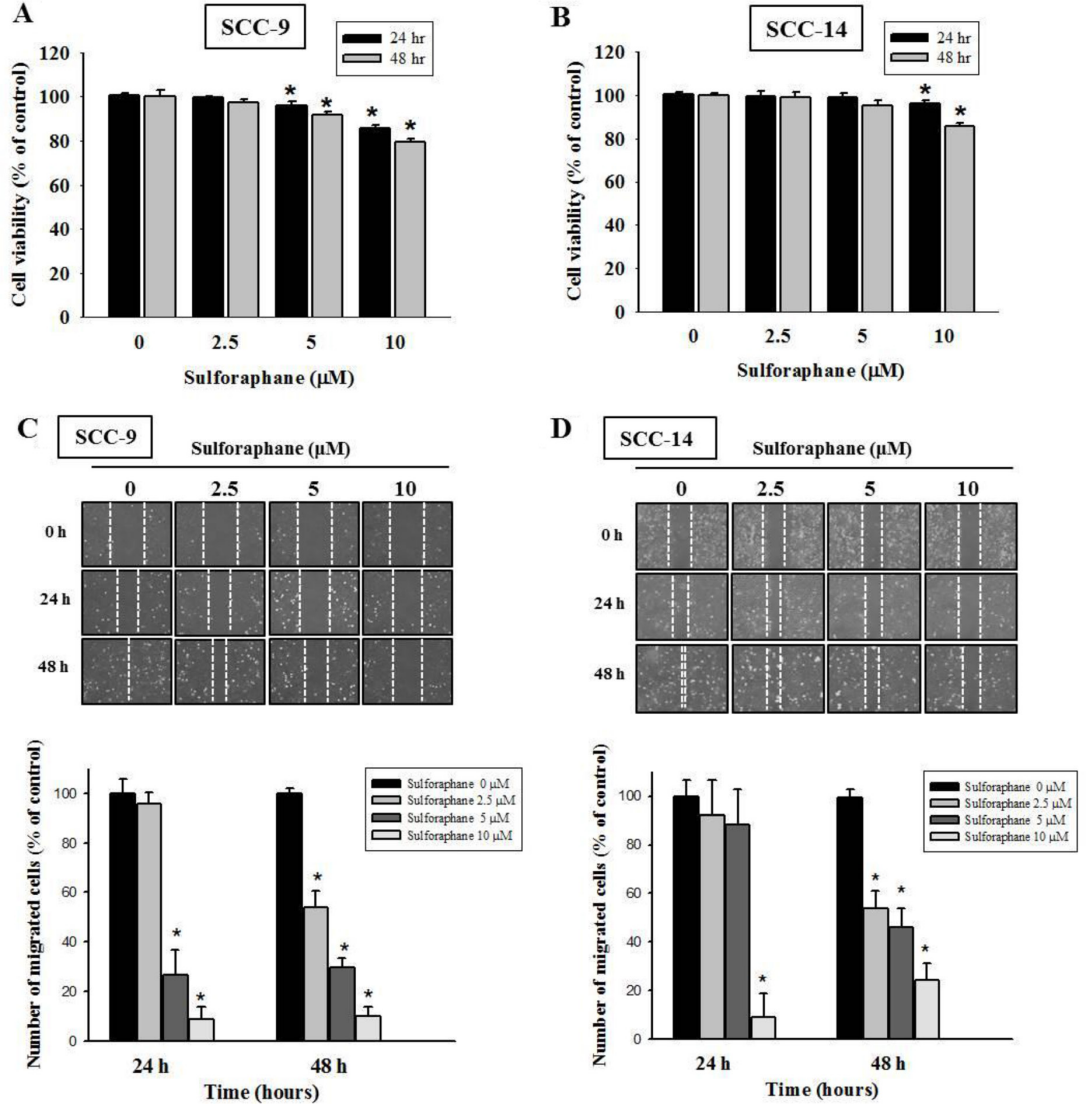
Effect of sulforaphane on cell viability and cell motility of SCC-9 and SCC-14 cells SCC-9 (**A**) and SCC-14 (**B**) cells were treated with different concentrations (0, 2.5, 5 and 10 μM) of sulforaphane for 24 h and 48 h before being subjected to an MTT assay for cell viability. The values represented the means ± SD of at least three independent experiments. ^*^p < 0.05, compared with the vehicle group. The motility of SCC-9 (**C**) and SCC-14 (**D**) cells were assessed by *in vitro* wound closure assay with different concentration of sulforaphane (0, 2.5, 5 and 10 μM) at different time points. A quantitative assessment of cell number in the denuded zone is the mean ± SD (*n* = 3). ^*^p < 0.05, compared with the vehicle group.

**Figure 2:**
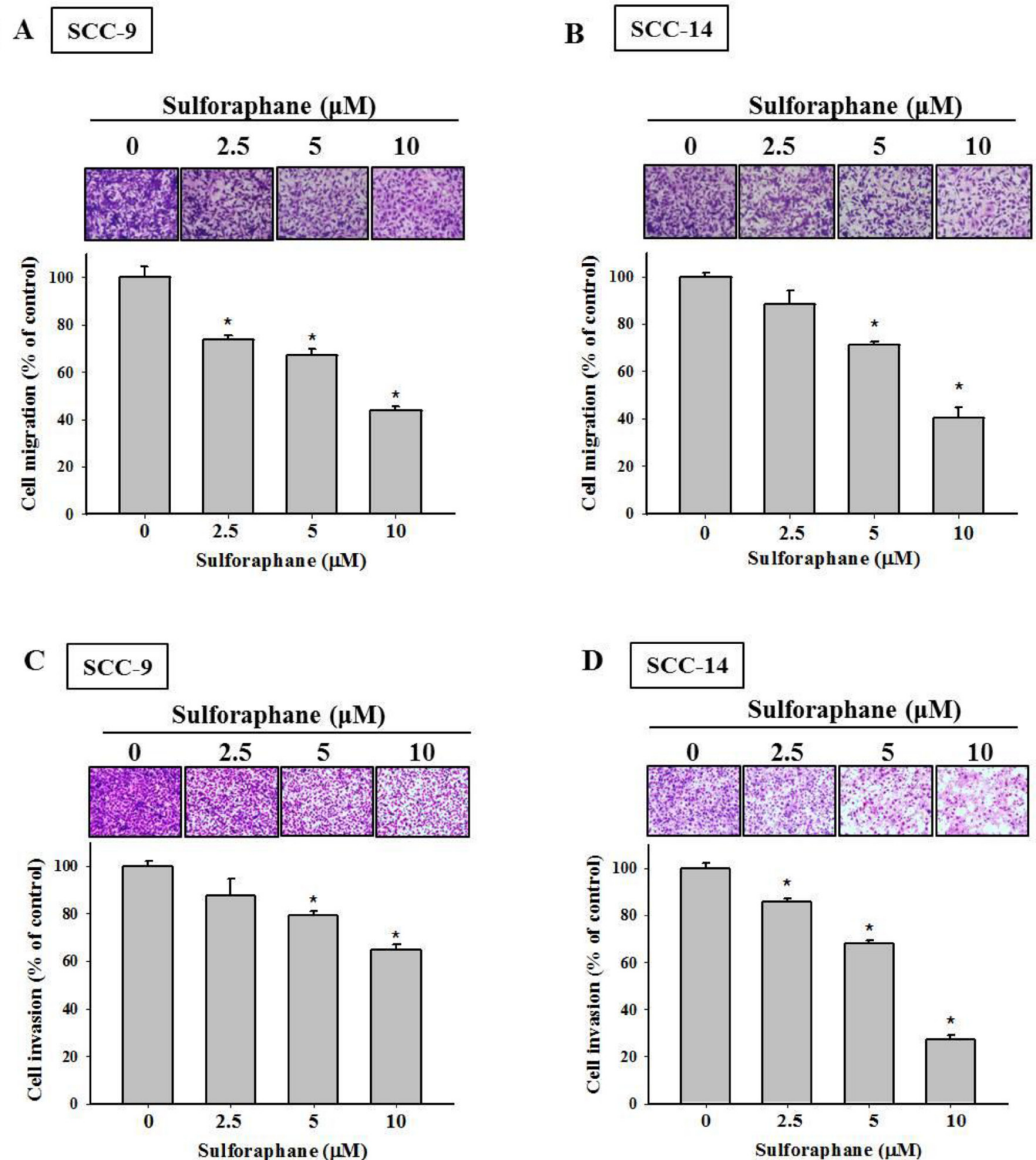
Effect of sulforaphane on cell migration and invasion of SCC-9 and SCC-14 cells The cell migration (**A**–**B**) and invasion (**C**–**D**) were measured using a Boyden chamber for 24 h and 48 h with polycarbonate filters. The number of cells which invaded the underside of the porous polycarbonate was counted for assessing the migration and invasion abilities of SCC-9 (A, C) and SCC-14 (B, D) cells. The values represented the means ± SD of at least 3 independent experiments. ^*^p < 0.05, compared with the vehicle group.

To investigate the effects of sulforaphane treatment on the viability of SCC-9 and SCC-14 oral cancer cells, an MTT assay was performed. Sulforaphane was administered to SCC-9 and SCC-14 cells for 24 h and 48 h at various concentrations (0, 2.5, 5 and 10 μM), as shown in Figure 1A and 1B. The data revealed that high-dose sulforaphane only reduced cell viability by 18% in SCC-9 cells after 48 h treatment. We further detected the migratory ability of SCC-9 and SCC-14 cells after sulforaphane treatment using a wound healing assay. Figure 1C and 1D show representative photographs of sulforaphane-reduced migration of SCC-9 and SCC-14 cells at 24 h and 48 h, respectively. To further investigate the effects of sulforaphane on cell migration and invasion, cells of both types were treated with or without sulforaphane for 24 h and 48 h, and samples were seeded in the upper chambers of an uncoated or Matrigel-coated filter. The same results were obtained; the numbers of both migratory and invasive cells were reduced when SCC-9 and SCC-14 cells were treated with sulforaphane (Figure 2A–2D).

### Cathepsin S expression was downregulated after treatment with sulforaphane

**Figure 3:**
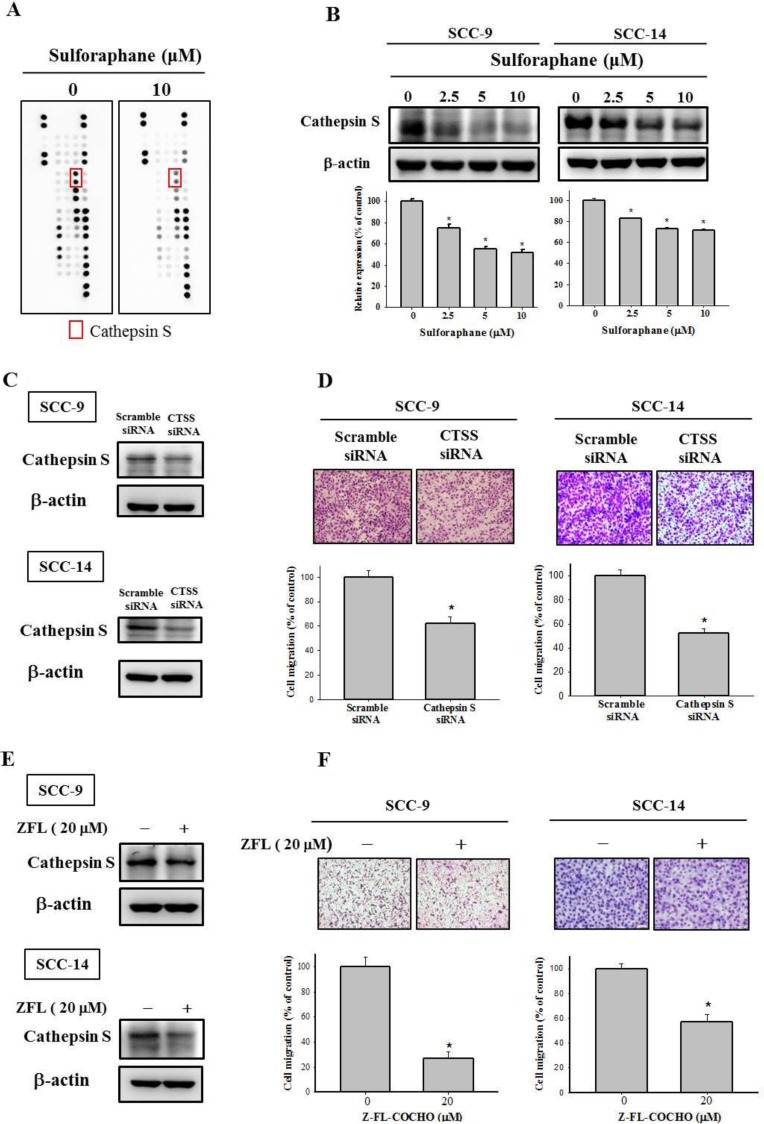
Effect of sulforaphane on the protein expression of cathepsin S of SCC-9 and SCC-14 cells (**A**) The cell protein lysate was collected and detected the protein expression by Human Proteinases Array. (**B**) The expressions of cathepsin S protein on the treatments of SCC-9 and SCC-14 cells were assessed by western blot. The values represented the means ± SD of at least 3 independent experiments. (**C**–**D**) SCC-9 and SCC-14 cells transfected with the siRNA of cathepsin S for 48 h and analyzed by western blot and Boyden chamber migration assay. (**E**–**F**). The cells were treated with cathepsin S inhibitor Z-FL-COCHO (20 μM) for 24 h then analyzed by western blot and Boyden chamber migration assay. The values represented the mean ± S.D. from 3 determinations per condition repeated 3 times. ^*^p < 0.05, compared with the vehicle group.

Because the dysregulation of proteinase expression is common in cancer progression, we used a human proteinase array kit to analyze the proteinase profile. As shown in Figure 3A, the protein expression of cathepsin S was lower in SCC-9 cells treated with sulforaphane at 10 μM than in the control group. In addition, we used Western blotting to confirm the cathepsin S protein expression; the data indicated that cathepsin S expression was reduced in both SCC-9 and SCC-14 cells (Figure 3B). Cathepsin S plays was demonstrated to play a vital role in numerous malignancies [7]. To repress cathepsin S activity, cathepsin S siRNA and an inhibitor of cathepsin S, Z-FL-COCHO (ZFL), were used in the subsequent experiments. The transfection and expression of cathepsin S–specific siRNA and the cathepsin S inhibitor significantly reduced cathepsin S protein expression in SCC-9 and SCC-14 cells (Figure 3C and 3E). Figure 3D and 3F both depict downregulated cathepsin S expression inhibiting the migration ability of SCC-9 and SCC-14 cells. These results indicate that sulforaphane treatment represses SCC-9 and SCC-14 cell migration by regulating cathepsin S expression.

### Effects of sulforaphane-induced autophagy and LC3 expression

**Figure 4:**
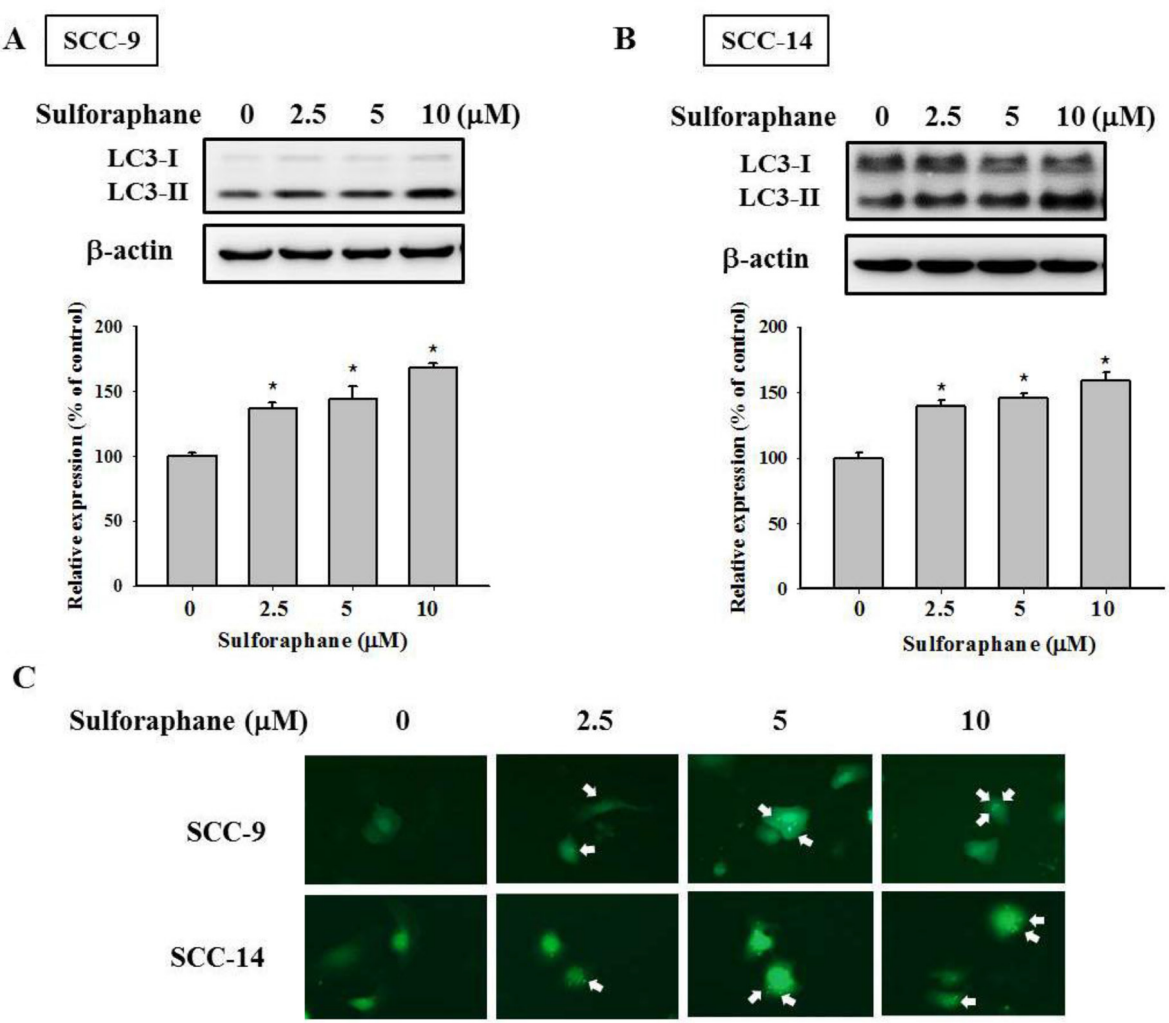
Effect of sulforaphane on the LC3 expression of SCC-9 and SCC-14 cells The expressions of LC3 protein on the treatments of (**A**) SCC-9 and (**B**) SCC-14 cells were assessed by western blot. The values represented the means ± SD of at least three independent experiments. ^*^p < 0.05, compared with the vehicle group. (**C**). GFP-LC3 dots were observed after the cells transfected with the plasmid and sulforaphane treatment.

**Figure 5:**
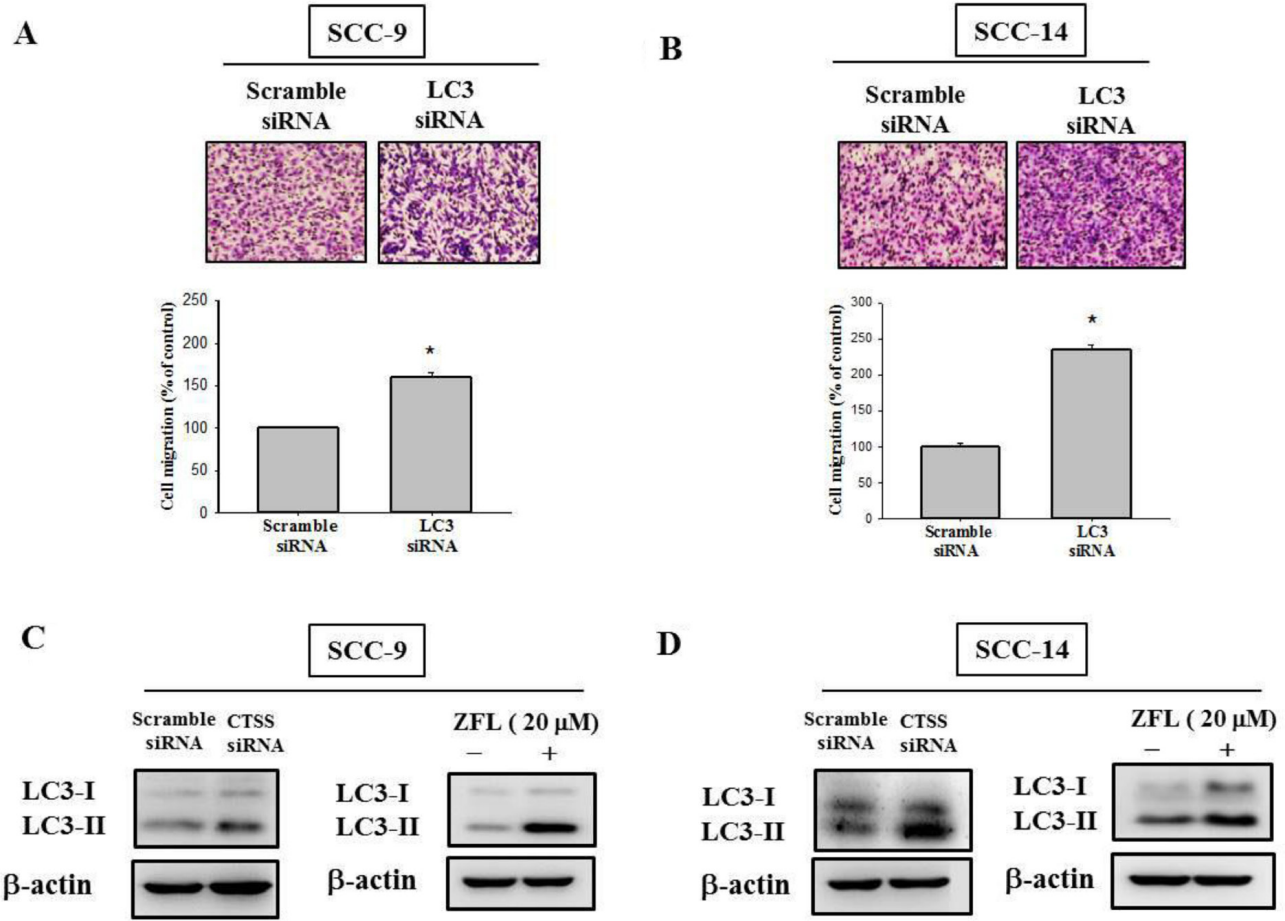
Effect of LC3 siRNA on the cell migration of SCC-9 and SCC-14 cells (**A**) SCC-9 and (**B**) SCC-14 cells transfected with the siRNA of LC3 for 48 h and analyzed by Boyden chamber migration assay. The values represented the mean ± S.D. from 3 determinations per condition repeated 3 times. ^*^p < 0.05, compared with the vehicle group. The (**C**) SCC-9 and (**D**) SCC-14 cells were treated with cathepsin S siRNA and cathepsin S inhibitor Z-FL-COCHO (20 μM) for 24 h then analyzed by western blot.

Relevant literature indicates that targeting cathepsin S induces tumor cell autophagy and increases microtubule-associated protein 1 light chain 3 (LC3) protein expression [12, 28]. During autophagy, the cytoplasmic form (LC3-I) is cleaved and lipidated to form LC3-II. To investigate whether autophagy plays a role in sulforaphane treatment of SCC-9 and SCC-14 cells, LC3 expression was examined. As shown in Figure 4A and 4B, LC3-II expression was elevated after sulforaphane treatment in SCC-9 and SCC-14 cells. Microscopy-based green fluorescent protein (GFP)-LC3 puncta formation assays were performed to confirm the aforementioned observation. The higher the concentration of sulforaphane treatment was, the greater the density of GFP-LC3 puncta in SCC-9 and SCC-14 cells (Figure 4C). To determine the relationship between LC3 expression and migration ability, we transfected SCC-9 and SCC-14 cells with LC3 siRNA. Knockdown of LC3 by siRNA increased the migration ability in both cell lines (Figure 5A and 5B). To explore whether cathepsin S regulates LC3 expression, cathepsin S siRNA and ZFL were used. As shown in Figure 5C and 5D, treatment of cells with cathepsin S siRNA and ZFL increased LC3-II expression in both SCC-9 and SCC-14 cells. Taken together, these results suggest that sulforaphane upregulates LC3-II expression by regulating cathepsin S expression, which results in the inhibition of cell migration.

### Sulforaphane alters levels of proteins associated with migration

**Figure 6:**
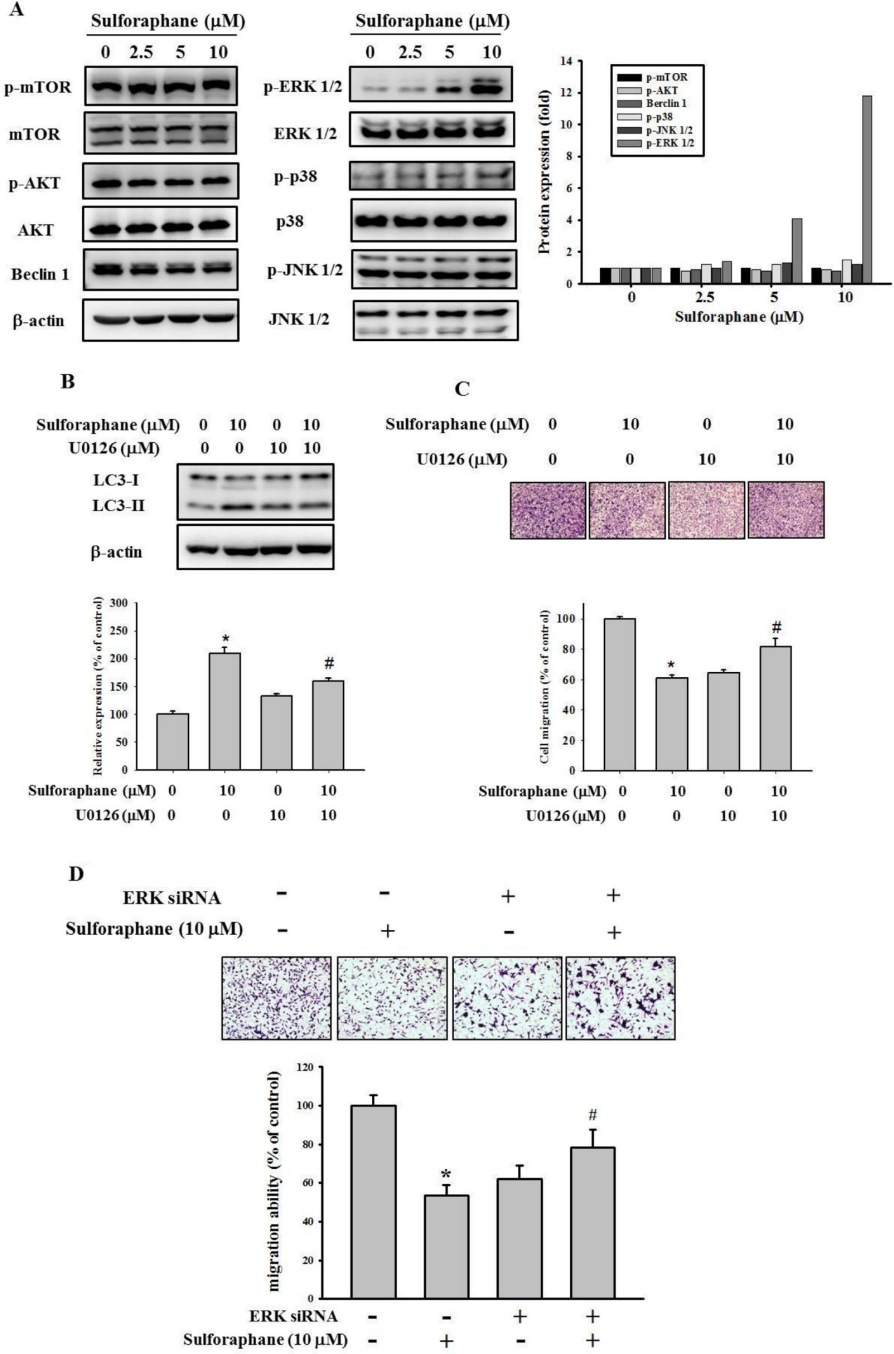
Effect of sulforaphane on the mTOR/AKT and MAPKs pathway (**A**). After a 24 h culture in various concentrations of sulforaphane (0, 2.5, 5 and 10 μM) for 24 h, the lysates of SCC-9 cells were subjected to SDS-PAGE followed by western blots with anti-mTOR, anti-AKT, anti-Beclin 1, anti-ERK, anti-JNK and anti-p38 (total and phosphorylated) antibodies. (**B**–**C**). SCC-9 cells were pretreated with or without U0126 (10 μM) for 1 h and then incubated in the presence or absence of combined sulforaphane (10 μM) for 23 h. The weastern blot assay was used for measurement of LC3 expression. Analysis of (C) cell migration of SCC-9 cells were assessed as described in Methods Section. (**D**) SCC-9 cells were pretreated with or without ERK siRNA (80 pmole) for 24 h and then incubated in the presence or absence of combined sulforaphane (10 μM) for 24 h. The values represented the means ± SD of at least 3 independent experiments. ^*^p < 0.05, compared with the vehicle group. ^#^*p* < 0.05, compared with the sulforaphane treated group.

To investigate the molecular mechanism of cathepsin S inhibition by sulforaphane, we examined the mTOR/AKT and MAPK signaling pathways, which are implicated in autophagy. The data showed that sulforaphane treatment did not affect AKT/mTOR or beclin 1 pathway expression (Figure 6A). However, sulforaphane obviously elevated the phosphorylation of ERK1/2. In addition, combined treatment with U0126, a MEK inhibitor, and sulforaphane reversed the inhibition of LC3-II conversion and migration by treatment with sulforaphane alone (Figure 6B and 6C). Moreover, combined treatment with ERK siRNA and sulforaphane also reversed the inhibition of cell migration by treatment with sulforaphane alone (Figure 6D).Taken together, these results suggest that sulforaphane represses cathepsin S by upregulating LC3-II expression; however, this occurs independently of mTOR/AKT-induced autophagy, at least mediating MAPK signaling pathways.

## DISCUSSION

Natural dietary agents comprise various types of biologically active compounds, including polyphenols, alkaloids, and isothiocyanates, that may provide abundant health benefits beyond basic nutrition. Research over the last decade has shown that dietary components in vegetables and fruits repress cancer growth [29]. Sulforaphane, a member of the isothiocyanate family, is abundant in widely consumed cruciferous vegetables and has potential anticarcinogenic properties. Early research focused on the inhibition activity of enzymes involved in carcinogen activation by sulforaphane [30]. However, more recent studies have demonstrated that sulforaphane offers protection against cancer development, through mechanisms including apoptosis induction, cancer stem cell proliferation, and autophagy modulation. In the present study, we demonstrated that sulforaphane inhibits cell migration by regulating cathepsin S and expression of its downstream target LC3.

Cathepsin S is a member of the cathepsin family that is predominantly expressed in antigen-presenting cells [31]. However, cathepsin S is also expressed in epithelial cells and various malignant tumor cells. Further insight into the roles of cathepsin S in tumorigenesis has been provided by the upregulation of cathepsin S in various tumor tissues [32, 33]. In addition, studies have indicated the significance of the relationship between cathepsin S and autophagy [11, 12, 28, 34, 35]. Zhang *et al.* revealed that the inhibition of cathepsin S induces autophagy through ROS-mediated PI3K/AKT/mTOR/p70S6K pathways [11]. Targeting cathepsin S induces autophagy and the conversion of LC3-I into LC3-II [12]. Lipidated LC3 is a useful marker of autophagic membranes. Our data clearly indicated that sulforaphane treatment can suppress the expression of cathepsin S and upregulate the expression of LC3-II (Figures 3A, 4A, and 4B). In addition, we discovered that knockdown of LC3 increased migration ability compared with that in the control group (Figure 5A and 5B). These results are in agreement with data reported by Zhao *et al.* [36], which demonstrated that low expression of LC3 contributed to a more aggressive cancer cell phenotype. In this study, targeting cathepsin S induced the conversion of LC3-I into LC3-II. In line with this finding, Chen *et al.* indicated that the downregulation of cathepsin S resulted in an increase of LC3-II and activation of the EGFR-related ERK/MAPK signaling pathway [12]. Mounting evidence indicates that the regulation of autophagy is complicated and several pathways are involved [37]. The AKT/mTOR and beclin 1 pathways have been revealed to be major regulators of the autophagic process [38, 39]. Nevertheless, our data indicated that the phosphorylation of AKT and mTOR, as well as the expression of beclin 1, is not affected by sulforaphane treatment (Figure 6A). The beclin 1-independent pathway was determined to be a noncanonical autophagy pathway in human breast cancer cells treated with resveratrol [40]. Kang *et al.* indicated that trehalose-induced autophagy is independent of mTOR and ROS in human podocytes [41]. Hence, much research remains to be conducted to discern the precise mechanism underlying sulforaphane treatment. In addition, several pathways are involved in the regulatory mechanism of the inhibition of migration by sulforaphane. Our results indicated that sulforaphane induced ERK1/2 phosphorylation. These data are consistent with those reported in the previous studies; sulforaphane triggered the ERK1/2 signaling pathway by increasing ROS [42, 43]. Moreover, Mondal *et al.* also stated that sulforaphane inhibits gastric cancer cell migration via EGFR and ERK1/2 pathway [44]. Our previous study demonstrated that hispolon suppresses metastasis through the autophagic degradation of cathepsin S with the activation of ERK in cervical cancer cells [28]. Additionally, the MEK inhibitor U0126 was used to show that sulforaphane inhibits cell migration through ERK activation (Figure 6C). Furthermore, Wang *et al.* suggested that sulforaphane inhibits thyroid cancer cell growth and invasiveness by enhancing p21 expression through the activation of ERK signaling cascades [45].

Collectively, our data provide evidence that sulforaphane inhibits human oral cancer cell migration by suppressing cathepsin S and the downstream target protein LC3. Furthermore, the ERK/MAPK pathways are involved in the regulation of oral cancer cell treatment with sulforaphane. Our findings suggest that cathepsin S and LC3 may be novel targets for oral cancer treatment.

## MATERIALS AND METHODS

### Material and chemicals

Sulforaphane was purchased from Sigma-Aldrich (St. Louis, MO) and the percent purity was over 90% determined by HPLC. Z-FL-COCHO was purchased from Calbiochem/EMD Millipore (Billerica, MA). MEK inhibitor U0126 was purchased from Promega (Heidelberg, Germany).

### Cell lines and culture

SCC-9 (human tongue squamous cell carcinoma, 25 year old male) obtained from ATCC (Manassas, VA, USA). SCC-14, a human floor of mouth squamous cell carcinoma cell line was purchased from CLS Cell Line Service GmbH (Eppelheim, Germany). Both of the cell lines cultured in DMEM/F-12 medium, 10% and 5% fetal bovine serum, respectively, 2 mM glutamine, 400 ng/ml hydrocortisone, 100 U/ml penicillin, and 100 μg/ml streptomycin (Sigma). The cultures were maintained at 37° C in a humidified atmosphere of 5% CO2.

### Cell viability assay

SCC-9 and SCC-14 cells were plated at a density of 8 × 10^4^ and 1.1 × 10^5^ cells/well in 24-well plates and treated with sulforaphane (0, 2.5, 5 and 10 μM) at 37° C for 24 h and 48 h , respectively. To determined cell viability, a microculture tetrazolium dye MTT (3-(4,5-dimethylthiazol-2-yl)-2,5-diphenyltetrazolium bromide) assay was performed, as described [46].

### Wound healing assay

SCC-9 and SCC-14 cells were grown onto 6-well plates and incubated overnight. Then the cells were scratched a line with pipette tips then treated with different concentrations of sulforaphane. We detected the ability of cell healing on different times by microscopy and measured the change of the mean number of cells from 3 replicates.

### Cell migration and invasion assay

The migration assay of SCC-9 cells was performed using Boyden chamber (Neuro Probe, Cabin John, MD, USA) for 24 h as described by Chung *et al* [47]. SCC-14 cells were seeded on Transwell inserts (Millipore, Bedford, MA, USA) at 10^4^ cells/well in serum-free medium and placed in the upper chamber then incubated for 48 h. For invasion assay, 10 μl Matrigel (25 mg/50 ml; BD Biosciences, MA, USA) was applied to polycarbonate membrane filters and the bottom chamber contained standard medium. The cell migratory abilities were determined by counting the migrated cells in five fields under high magnification.

### Western blot assay

SCC-9 and SCC-14 cells were seeded 6 × 10^5^ and 1.2 × 10^6^ onto 6 cm dish, respectively, and treated with sulforaphane. Cell lysate were collected with 50–100 μL of protein extraction solution (iNtRON Biotechnology, Seongnam, Korea) as previously described [48]. After centrifuged at 13000 g at 4° C for 30 min. The protein lysate were separated by 12% agarose gel and transferred onto a nitrocellulose membrane then blocking with 5% non-fat milk in Tris-buffered saline (20 mM Tris, 137 mM NaCl, pH 7.6) for 1 h in room temperature and overnight with first-antibodies in 4° C and second-antibodies for 1 h in room temperature. Anti-p-ERK, anti-ERK, anti-JNK, anti-p-JNK, anti-p-Akt, anti-LC3I, anti-Beclin 1, anti-p-mTOR, anti-mTOR were purchased from Cell Signaling Technology (Danvers, MA, USA). Anti-Akt, anti-pp38, anti-p38 were purchased from BD Biosciences (Bedford, MA, USA). Anti-cathepsin S was purchased from GeneTex (CA, USA). The band intensities were quantified by densitometry.

### GFP-LC3 transfection and GFP-LC3 dot formation

The cells were culture overnight then transfected with GFP-LC3 as previously described [28]. After incubated for overnight, the cells were treated with sulforaphane (0–10 μM) for 24 h. The GFP-LC3 punctated dots in cells were obtained using fluorescence microscope.

### Small interfering RNA (siRNA) system

siRNA oligonucleotide duplexes targeting human cathepsin S and LC3B were purchased from Invitrogen (Carlsbad, CA). SCC-9 and SCC-14 cells were seeded 6 × 10^5^ and 1.2 × 10^6^ onto 6 cm dish, respectively. After cultured for 24 h, cells were transfected with siRNA for 48 h using Lipofectamine 2000 (Invitrogen) according to the manufacture’s instructions.

### Statistical analysis

The statistical analysis was performed using the Student’s *t*-test (SPSS, Chicago, IL, USA). Significance was set at *p* < 0.05. The values are the means ± standard deviation (SD) of at least three independent experiments.
